# Exploring yeast interactions through metabolic profiling

**DOI:** 10.1038/s41598-020-63182-6

**Published:** 2020-04-08

**Authors:** C. Roullier-Gall, V. David, D. Hemmler, P. Schmitt-Kopplin, H. Alexandre

**Affiliations:** 1 UMR PAM Université de Bourgogne/AgroSup Dijon, Institut Universitaire de la Vigne et du Vin, Jules Guyot, Dijon, France; 2 Comprehensive Foodomics Platform, Technische Universität München, Freising, Germany; 3 Research Unit Analytical BioGeoChemistry, Department of Environmental Sciences, Helmholtz Zentrum München, Neuherberg, Germany

**Keywords:** Metabolomics, Small molecules, Chemical biology, Microbiology, Plant sciences, Mass spectrometry

## Abstract

As a complex microbial ecosystem, wine is a particularly interesting model for studying interactions between microorganisms as fermentation can be done by microbial consortia, a unique strain or mixed culture. The effect of a specific yeast strain on its environments is unique and characterized by its metabolites and their concentration. With its great resolution and excellent mass accuracy, ultrahigh resolution mass spectrometry (uHRMS) is the perfect tool to analyze the yeast metabolome at the end of alcoholic fermentation. This work reports the change in wine chemical composition from pure and mixed culture fermentation with *Lachancea thermotolerans*, *Starmerella bacillaris*, *Metschnikowia pulcherrima* and *S. cerevisiae*. We could clearly differentiate wines according to the yeast strain used in single cultures and markers, which reflect important differences between the yeast species, were extracted and annotated. Moreover, uHRMS revealed underlining intra species metabolomics differences, showing differences at the strain level between the two *Starmerella bacillaris*. Non volatile metabolomics analysis of single and sequential fermentations confirmed that mixed fermentations lead to a different composition. Distinct metabolites appeared in wines from sequential fermentation compared to single fermentation. This suggests that interactions between yeasts are not neutral.

## Introduction

Microorganisms coexist in most environments and interact with each other. These interactions happen in nearly every niche on the planet and in numerous processes such as bioremediation of pollutants, farming, biotechnology, medicine or food-processing^1,2^. Under oenological conditions, when yeasts grow simultaneously during alcoholic fermentation, they often do not coexist passively, and in most cases, physiological and metabolic interactions are established between them. The interactions between the different strains of *Saccharomyces* and non-*Saccharomyces* yeast can be direct or indirect, through the physicochemical changes in the environment caused by one strain reacting to the other. In oenology, the effect of these interactions is characterized as being positive, negative or neutral^3^. Genomics and proteomics provide an understanding of the interaction^3–5^ but none of them could yet provide significant progress on the microbial interaction mechanisms. Recently, high resolution mass spectrometry has been used to elucidate interactions between yeasts and bacteria in the malolactic fermentation by comparison of extracellular metabolic profiles^6^. Even more recently, ultrahigh resolution mass spectrometry (uHRMS) confirmed that cell-cell contact influences the metabolism of *L. thermotolerans* and *S. cerevisiae*^7^. Therefore, metabolomics seems to be a suitable tool to better understand the microbial interactome in order to control fermentation by multi-starters. Metabolomics is defined as the study of all metabolites given in a biological system under particular physiological conditions^8^. The analytical techniques developed for metabolomics studies allow the screening of hundreds of metabolites from complex biological samples with high-throughput^9,10^. Therefore, the level of metabolites represents integrative information of the functional status of the cell and defines the phenotype of a cell in response to genetic or environmental changes^11^.

The chemical composition of wine can be described as a complex product integrating signatures from many factors, including grape variety, geographical origin, viticultural condition of grape cultivation, microbial ecology of the grape, fermentation process and winemaking practices^12^. Yeasts, bacteria and filamentous fungi all contribute to the chemical composition of wine. Among these, yeasts have the highest influence because of their role in conducting the alcoholic fermentation^13^. Although *Saccharomyces* species are the main fermenting yeast strains other non-*Saccharomyces* yeasts can be responsible for alcoholic fermentation^14^. In all fermented products the microbiota contribute to a large extent to the development of the typical color, flavor, and texture of the final product^15^. Within the great diversity of yeast types, non-*Saccharomyces* are more and more studied because of their capability to improve the complexity of the wine aroma by increasing the concentration of specific aromatic molecules, terpenoids, higher alcohols^16–21^ or glycerol^22,23^.

Interactions between strains *of Saccharomyces cerevisiae* have been demonstrated by Howell *et al*.^24^. In an original and very interesting way, this study highlights that the wines produced by assembling wines from pure cultures had a different composition of volatile compounds compared to wines produced by co-cultivation. This study showed that within the same species different strains interact and that these interactions have an impact on the composition of the final wine sensory profile. King *et al*.^25^ and Capece *et al*.^26^ conducted similar studies and confirmed that *Saccharomyces cerevisiae* co-cultures differ from single cultures in the sensory profile of the final product but also differ from the wine assemblages of each mono-culture. However, the nature of the interactions and mechanisms that regulate microbial interactions, population composition, dynamics, and performance remain largely elusive.

In recent years, “omics”-technologies were used to discover new aspects of microbial interactions. For instance, transcriptomic analysis combined with physiological data provided an integrated view into the response of a yeast to the environment during mixed culture fermentation^4^. Moreover, investigation of the behavior of strains in mixed cultures suggests that cell-cell contact and aggregation are indispensable to gain the dominance of one strain over another^27^. Likewise, after 65 generations, co-evolved strains and strains evolved independently show heritable variation in growth as well as flavor and aroma profiles^28^. Additionally, metabolic footprints of multiple yeast strains in monocultures, mixed cultures or blended samples showed differences in the sensory profile, which highlights the impact of one strain on the metabolic behavior over others^24,29^. Moreover, NMR-based metabolomics was recently used to identify metabolites that discriminate single and mixed cultures of two yeast during fermentation^30^. However, reciprocal responses of both microorganisms have not been studied.

More and more work focused in the study of volatile metabolome and inter-strains variability^31–35^ but very few have been interested in non-volatile composition. In this study, we aimed to characterize different non-*Saccharomyces* yeast species and to compare their non-volatile metabolic fingerprint to the most frequently used species in winemaking, i.e. *Saccharomyces cerevisiae*. For this purpose, we used three different *non Saccharomyces* (NS) species, *L. thermotolerans* (LT), *S. bacillaris* (SB1 and SB2) and *M. pulcherrima* (Mp) that have been reported to complete alcoholic fermentation^7,14,36^ and one *Saccharomyces cerevisiae* strain (Sc). It has been previously shown that interactions between yeasts in sequential cultures lead to wines that differ from wines with single cultures in their volatile compounds^14,37^. Then, another goal was to study the influence of these non-*Saccharomyces* strains in sequential cultures with *Saccharomyces cerevisiae* on the non-volatile wine metabolome.

## Results and discussion

**Figure 1:**
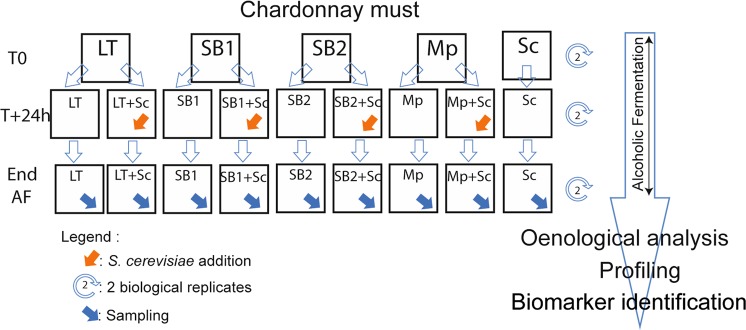
Experimental workflow. Legend: *Lachancea thermotolerans* (LT), *Starmerella bacillaris* (SB1 and SB2), *Metschnikowia pulcherrima* (Mp) and *Saccharomyces cerevisiae* (Sc).

Chardonnay must was divided into 10 aliquots, which were inoculated with pure cultures of *S. cerevisiae* (Sc), *L. thermotolerans* (LT), *S. bacillaris* (SB1 and SB2) and *M. pulcherrima* (Mp), respectively. After 24 h, Sc was added to five of the aliquots (Fig. 1) to promote the impact of non-*Saccharomyces* yeasts^38^. At the end of the alcoholic fermentation, all samples from sequential fermentation had an ethanol concentration of about 10% (*v/v*) and a sugar concentration lower than 2 g.L^−1^. Therefore, differences in the final wine compositions are characteristic to the impact of the yeast or the mixture of yeasts used for the fermentation.

### Impact of the type of yeast on the exometabolome

A visual comparison of FT-ICR-MS raw spectra of Chardonnay wines, which only differ in the yeast used for the alcoholic fermentation (Sc, LT, SB1, SB2 and Mp) is presented in the supplemental Fig. 1. The impact of yeast strains on chemical spaces of wine can already be observed in the mass distributions of spectra from the different wine samples. Several thousands of signals were found in the mass range of *m/z* 100 to 1000. For example, signals at *m/z* 259.01291 and *m/z* 259.02243, corresponding to the molecular formulae [C_6_H_11_O_9_S]^−^ and [C_9_H_11_N_2_O_3_S_2_]^−^, respectively, showed strong differences in their ion abundances (Supplemental Fig. 1B). Moreover, signals at *m/z* 267.03576 and *m/z* 267.07214, corresponding to the molecular formulae [C_8_H_11_O_10_]^−^ and [C_9_H_15_O_9_]^−^, respectively, show an inverse behavior of intensities between Sc and non-Sc yeast strains (SB1, SB2, Mp and LT).

**Figure 2:**
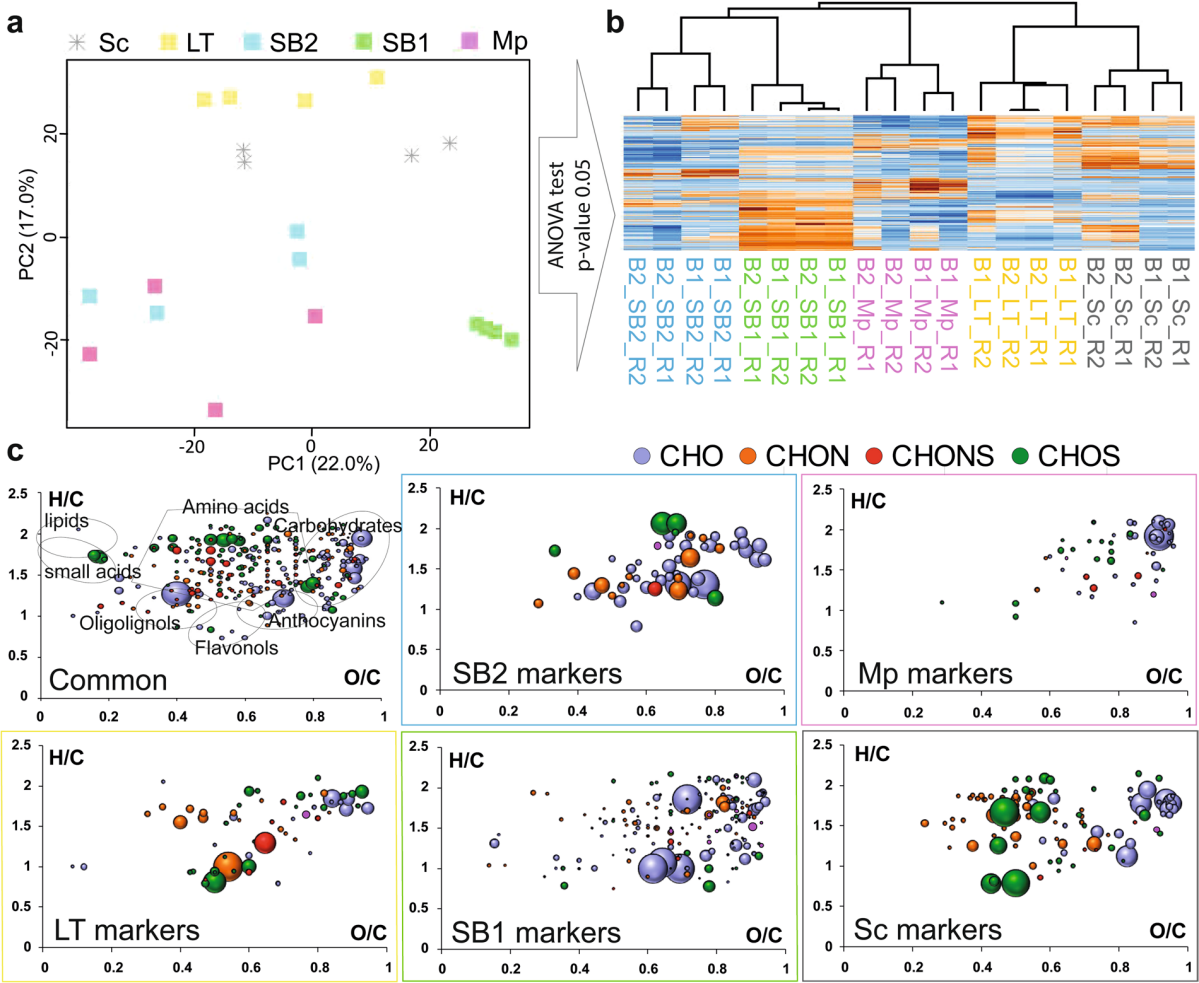
(**a**) Principal component analysis of single yeast fermentations using direct methanol dilution. The first two components represent 39% of the variability. (**b**) ANOVA statistics and hierarchical cluster analysis were used to extract specific masses for each yeast strain. (**c**) Van Krevelen diagrams show elemental compositions common and specific to all analyzed yeast strains. Bubble sizes indicate relative intensities of corresponding peaks in the spectra. Color code: CHO, blue; CHOS, green; CHON, red; CHONS, orange.

The principal component analysis (PCA) of FT-ICR-MS raw data from the single-culture experiments (Fig. 2a and Supplemental Fig. 2) illustrates the repartition of samples based on their chemical composition. Biological and technical replicates of each wine appeared close in the PCA. This confirms good repeatability of the fermentation and analysis. Interestingly, using the first three components, wines were well separated from each other (the first three components explained 53.2% of the variability - Supplemental Fig. 2). SB1 (in green) could clearly be separated from other wines on PC1 (the first component explained 22.0% of the variability). By comparison, Sc-samples showed higher similarity to LT-samples but could be distinguished on PC2 (17.0% of variability). SB2- and Mp-samples appeared in an unresolved cluster in Fig. 2 but could be distinguished on PC3 (Supplemental Fig. 2). This confirms our hypothesis that different yeast strains impact the exometabolome fingerprints, which can be retrieved from uHRMS measurements. This powerful technique allows wines produced with different yeast strains or even with different species (i.e. SB1 and SB2 separated on PC1) to be distinguished. Indeed, the influence of these species in winemaking on some specific target compounds has already been studied^30,39–42^. NMR-based metabolomics was recently used in order to discriminate yeast cultures during alcoholic fermentations, but unfortunately, NMR was not able to fully discriminate all yeasts^30^.

In order to visualize and characterize the impact of yeasts on the final wine composition, we computed ANOVA statistics for all compounds detected by FT-ICR-MS. Consequently, we retrieved a subset of features that showed significant differences in their mean peak intensities (p < 0.05) between the different sample groups. These extracted features can be used as markers to distinguish and characterize the wine depending on the yeast used for the fermentation. The subset contained 336, 255, 572,102 and 234 possible markers specific for Sc, LT, SB1, SB2, and Mp yeasts, respectively (Fig. 2b). The number of markers extracted for each type of wine highlights the variable impact of each yeast strain onto the final wine composition. The highest number of markers was found for SB1 (572 specific compounds) which is in agreement with the results obtained by PCA (Fig. 2a). Conversely, SB2 wines showed the smallest number of significant markers (102) indicating that the SB2-specific impact on wine composition is smaller than that of other yeast species.

In addition to the specific markers, we found 40, 19, 28, 68 and 11 metabolites which could exclusively be detected in Sc, SB1, SB2, Mp, and LT samples, respectively. Moreover, metabolomics highlighted the diversity within species. Indeed, Fig. 2 clearly shows that the two *Starmerella bacillaris* (SB) strans differ in the produced metabolites. Our results confirmed that different NS species have a wide intra specific variety^43–46^.

Van Krevelen diagrams of the extracted markers illustrate the diversity of metabolites formed by the different yeast strains during fermentation (Fig. 2c). For example, Sc markers are mainly composed of CHO and CHOS compounds in the area of the van Krevelen diagram were carbohydrate- (O/C > 0.8 and 1.6 < H/C < 2.7), polyphenol- (0.4 < O/C < 0.7 and 0.5 < H/C < 1.5) and amino acid-derivatives (0.1 < O/C < 0.6 and 0.9 < H/C < 2.5) are expected^47^. By comparison, SB1 markers are mainly composed of CHO in the van Krevelen diagram are of O/C > 0.8 and 1.6 < H/C < 2.7 (potentially carbohydrate-type compounds). Possible structural assignments for compounds that discriminate the wine samples could be obtained from the literature and relevant databases (YMDB, KEGG, Metlin, Lipidmap). Only a low number of the detected molecular formulae led to possible structure assignments in the databases. We found 16 (4.7%), 8 (3.1%), 79 (13.8%), 14 (13.7%) and 17 (7.2%) possible metabolite markers for the Sc, LT, SB1, SB2, and Mp sample, respectively (Supplementary table 1). The low percentage of annotated markers illustrates the extent of the unknown composition of wine where at best for FT-ICR-MS data less than 20% of detected features can find hits in databases^48,49^.

As several possible isomers might exist for each of the signals detected in FT-ICR-MS experiments, we additionally performed LC-MS/MS experiments and used databases to confirm previous annotations. Out of the 134 markers reported above, in total 40 compounds could also be detected by LC-MS/MS. The lower coverage in LC-MS/MS compared to FT-ICR-MS is because of the lower sensitivity of the Q-ToF instrument^49,50^. MS/MS spectra obtained were manually extracted and compared to known or predicted MS/MS spectra from Metlin, Metfrag and HMDB databases. Ten structures could be confirmed based on MS/MS (supplementary table 2 and Supplementary Fig. 3). Surprisingly none of these 10 markers are directly produced by yeast during fermentation. For example, phenolic compounds are not metabolized by yeast during fermentation but can be adsorbed on the yeast cell wall^51,52^ and therefore may cause variation in fertaric acid or piceid levels identified as SB1 and SB2 markers, respectively. It has been reported that the adsorption capacity depends on the strain^53^ which might explain our observations. Another SB1 marker, identified as isopropylmalate, is an intermediate of leucine biosynthesis^54^. Identified markers for each species are not necessarily metabolized by yeast but may vary in intensity depending on the consumption or production of metabolic intermediates. These results suggest that, to date, a large part of the yeast-specific metabolites remain unidentified in the databases.

### Impact of mixed cultures on the exometabolome

In addition to single yeast fermentation, the must was fermented by LT, SB1, SB2 and Mp in sequential cultures with Sc (Fig. 1). A visual comparison of FT-ICR-MS raw data obtained from the Chardonnay wines after sequential fermentation is presented in Supplementary Fig. 4. Results of a principal component analysis (PCA) computed from data of all wines (pure culture and sequential fermentation) indicates that none of the sequential fermented wines show similar composition to the respective wine after single fermentation (Supplementary Fig. 5). According to the first component (21.1% of the variation), SB2 wines from pure (SB2) and co-fermentation (SB2 + Sc) are the two most discriminant groups of wine. SB1 in pure (SB1, pale green) and co fermentation (SB1 + Sc, dark green) are distinguished according to the second component (12.8% of the variation). Finally, PCA indicates a higher proximity of LT and SB1 in co-fermentation wines (LT + Sc in orange and SB1 + Sc in dark green) to Sc wines (in grey). This first result highlights the variable impact of Sc depending on the NS yeast used in the fermentation. The impact of NS on some specific target compounds, especially volatile compounds, has been reported^14,55^. By comparison, our results show the impact of sequential fermentation of *Saccharomyces cerevisiae* with non-*Saccharomyces* strains on the non volatile wine exometabolome.

**Figure 3:**
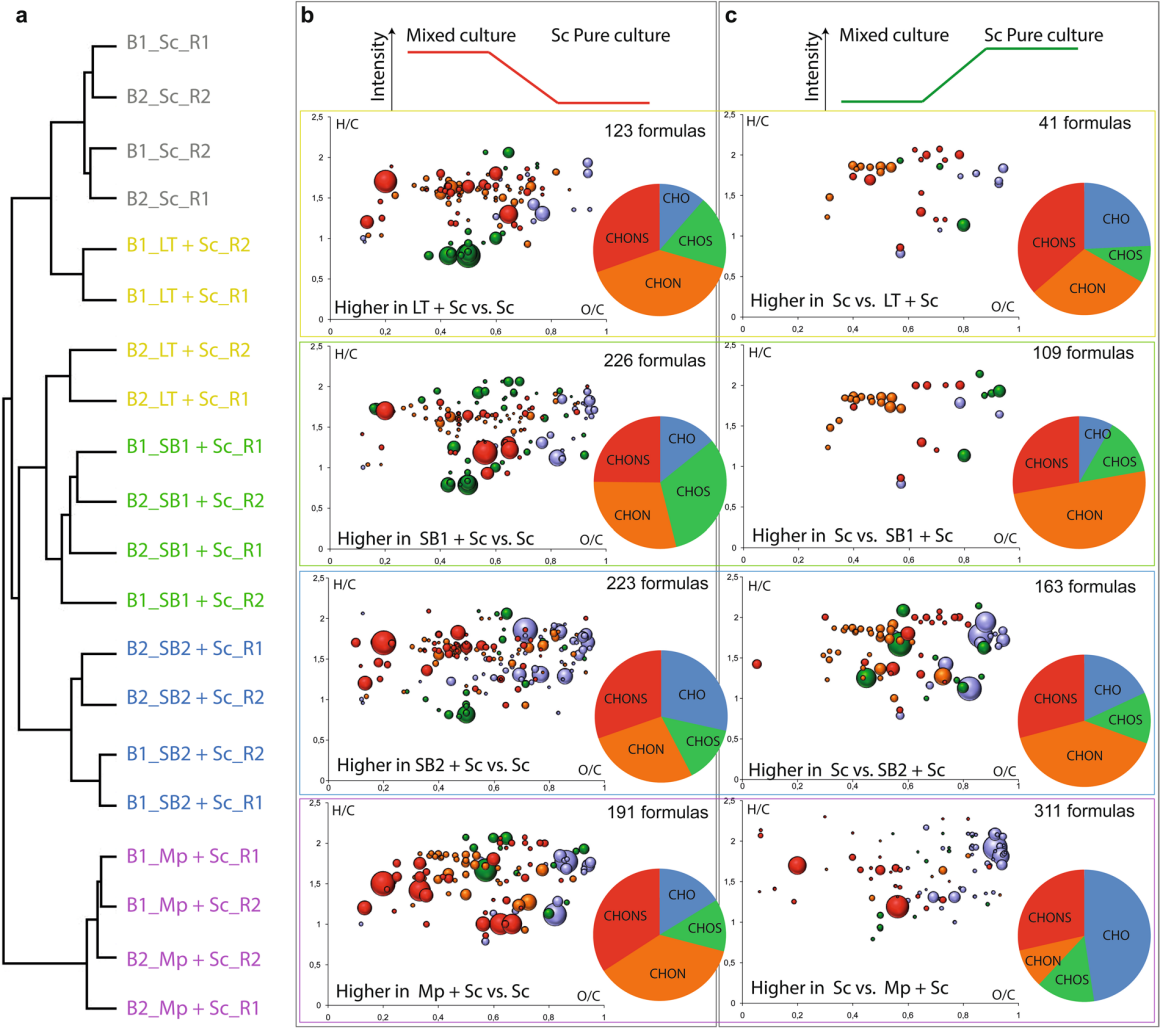
(**a**) Hierarchical cluster analysis of Sc alone together with all sequential fermentated samples: LT + Sc, SB1 + Sc, SB2 + Sc and Mp+Sc. Van Krevelen diagrams and ratio of the compositional families specifically show detected compositions with significantly higher (**b**) and lower (**c**) peak intensities in each sequential fermented sample compared to the Sc pure culture. Bubble sizes indicate relative intensities of corresponding peaks in the spectra. Color code: CHO, blue; CHOS, green; CHON, red; CHONS, orange.

The impact of Sc on the chemical composition seems to depend strongly on the type of NS yeast strains used in the sequential fermentation. To better understand the Sc impact in mixed-fermentation, we studied the evolution of the chemical composition between sequential culture and single yeast fermentation. Cluster analysis of wines from sequential fermentation experiments and from Sc alone confirmed that all sequentially fermented wines have a different chemical composition compared to Sc alone (Fig. 3a). Similar to the results in Fig. 2b, SB1 + Sc and SB2 + Sc build a cluster. Mp+Sc differs most from Sc wines according to the HCA in Fig. 3a. In order to investigate the contribution of Sc in sequential fermentations and the role of the NS yeast in the final wine composition, we extracted those features which showed a significant difference in peak intensities between the wines from Sc alone and from each sequential fermentation experiment (Fig. 3b,c). Interestingly, we found very specific elemental compositions for each of the analyzed yeast strains. For example, the presence of LT in LT + Sc wines is characterized by high number of CHNO (in orange) and CHNOS (in red) compounds in an area of the van Krevelen diagram where amino acid and polyphenol derivatives can be expected (Fig. 3b). The presence of SB1 in SB1 + Sc is characterized by a high number of CHOS (in green) directly followed by CHNO and CHNOS while the presence of SB2 in SB2 + Sc is characterized by high numbers of CHO (in blue), CHNO and CHNOS in an area of the van Krevelen diagram where amino acids, polyphenols and carbohydrates are usually found. The presence of Mp in Mp+Sc wines is different to other yeasts and characterized by a higher number of CHNO and CHNOS but mostly because of a reduction of CHO compounds in an area of the van Krevelen diagram where carbohydrates are usually found. According to these results, sequential fermentation leads to a different chemical composition than Sc alone which is in line with previous results based on targeted analysis of mainly volatile compounds^30,56^. The type of yeast impacts the final wine composition and in the case of sequential fermentations, the presence of a NS yeast modulates the wine compositional diversity.

**Figure 4:**
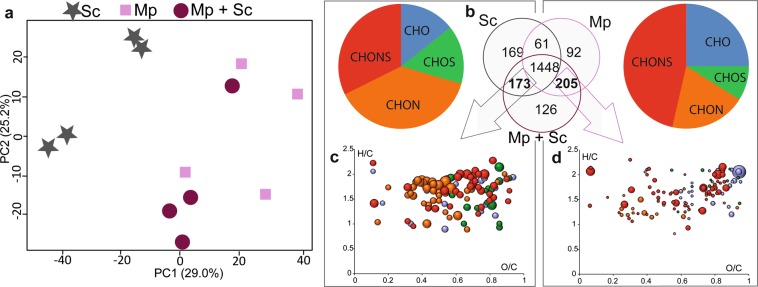
(**a**) Principal component analysis of Sc and Mp single fermentations and Mp + Sc mixed fermentation with (**b**) Venn diagrams. Common markers of (**c**) Sc and Mp + Sc and (**d**) Mp and Mp + Sc are highlight in the van Krevelen diagrams and histograms representating the molecular formulas composition. Bubble sizes indicate relative intensities of corresponding peaks in the spectra. Color code: CHO, blue; CHOS, green; CHON, red; CHONS, orange.

In a next step, we compared and characterized the non volatile chemical composition between the wines produced from single-yeast fermentation and the corresponding sequential fermentation (Fig. 4 and Supplementary Fig. 6). The PCA shows that SB2 + Sc and Mp+Sc have a composition which is more similar to the corresponding NS wines (SB2 and Mp, respectively) than to the Sc wines. By comparison, LT + Sc and SB1 + Sc indicate higher similarity to Sc than NS wines (LT and SB1, respectively). This is in agreement with the result shown in Fig. 3 and highlights the higher impact of Sc on the final wine composition. On the opposite, the higher proximity of the sequentially-fermented wines Mp-Sc and SB2-Sc compared to the single NS wines (Mp and SB2, respectively) illustrates the higher impact of NS on the final wine. The study of the four sequential fermentations illustrates perfectly the various possible interactions between Sc and NS, especially interesting is the reversed behavior of the two SB strains. Thus, the exometabolome reflects the greater or lesser dominance of one species compared to another. This aspect of dominance cannot be evaluated by targeted approaches.

It is very interesting to note that, despite the large number of common compounds found between single yeast fermentations and the corresponding sequential fermentation, Sc and each NS possess a certain number of unique compounds (Fig. 4 and Supplementary Fig. 6). The sequential fermentation appears not only as the simple addition of compounds from both fermentations because some compounds present in single yeast wines are not found in the corresponding sequential fermentation. Furthermore, some other compounds are unique to sequential fermentations. For example, for Sc and Mp fermentations, 169 compounds were unique to Sc (leading to 342 specific features for Sc), 92 unique to Mp (leading to 297 specific features for Mp), and 126 were only detected in the sequentially fermented wines (Mp + Sc) (Fig. 4b). Moreover, a part of the Sc specific composition (342 compounds only present in wine if Sc is used for fermentation) was also found after sequential fermentation (173 molecular formulae which are specific to Sc). This approach gives information on the nature of the interaction between the species. Indeed, it is clear from the results presented in Fig. 4 and Supplementary Fig. 6 that the interactions occurring between Sc and the three NS species are not neutral because, as stated above, new compounds arise from the sequential fermentation compared to single fermentation. The presence of new metabolites in sequential fermentation not detected in single cultures reflects positive or negative interactions between species^57–59^.

## Conclusion

Previous metabolome studies of wine from single and mixed cultures typically focus on targeted analysis and volatiles analysis by GC-MS^14,24,30,37^. Targeted analysis is commonly used to verify hypothesis whereas the non-targeted approaches allows generating new hypothesis. This work reports the changes of the non-volatile chemical composition of the wine exometabolome as triggered by fermentation in single and mixed culture using ultra high resolution mass spectrometry (uHRMS). Using uHRMS, like FT-ICR-MS, a wider range of metabolically relevant features can be detected. We confirm that tremendous differences exist between species and extract hundred of non volatile metabolites specific to the yeast or the mixed of yeast used during alcoholic fermentation. The presence of compounds which are specific to a yeast strain can be used to find the identity of yeasts involved in the fermentation. Another important aspect that stands out from our study is the significant contribution of non-*Saccharomyces* yeasts together with *Saccharomyces cerevisiae* to the non-volatile composition of wines. Indeed, while we clearly showed that single cultures could be easily discriminating from sequential cultures based on their metabolite profile, our data are consistent with the existence of positive or negative interactions between yeast species. In fact, the wine composition of sequential culture is not only the addition of metabolites from each species but is the result of complex interactions causing the presence or the absence of specifics compounds. The complexity of wine composition and yeast interaction require instrumental analytics and scientific methods that can detect, identify, quantify and characterize metabolite. uHRMS analysis may be used for understanding metabolic changes during wine fermentations where yeast cultures are used in sequential fermentation. However, information obtained using FT-ICR-MS is neither quantitative nor complete in terms of structure elucidation and improvement of metabolome databases would certainly allow to unravel how the metabolism of one species is modulated by the presence of another species.

## Materials and methods

### Yeast strains

Four non-*Saccharomyces* yeast strains were obtained from the collection of the Burgundy University Vine and Wine Institute. The strains selected for this study were *Lachancea thermotolerans* (LT), two strains of *Starmerella bacillaris* (SB1 and SB ∙ 2), *Metschnikowia pulcherrima* (Mp) and a commercial strain of S*accharomyces cerevisiae* (Sc) used as a reference and for sequential inoculation (Fig. 1).

All yeast strains were grown at the controlled temperature of 28 °C on modified yeast extract dextrose medium (YPD 20 g.L^−1^ glucose, 10 g.L^−1^ peptone, 5 g.L^−1^ yeast extract with 20 g.L^−1^ agar) and supplemented with 0.2 g.L^−1^ of chloramphenicol^7^. Yeasts were pre-cultured for 24 h in 250 mL sterile Erlenmeyer flasks, closed with dense cotton plugs, containing 150 mL of modified YPD medium under agitation (100 rpm)^7^.

### Enumeration of microorganisms

After yeast growth in YPD medium, 2 microtubes containing one milliliter of yeast culture with 10^6^ cells were centrifuged (9000 g for 5 min). The pellet was suspended in 1 mL MacIlvaine’s buffer (0.1 M citric acid, 0.2 M disodium hydrogenphosphate; pH 4). The first tube was used as a control. The second tube was complemented with 2 µL of the viability probe 5-CFDA, AM (5-carboxyfluorescein diacetate, acetoxymethyl ester; Thermo Fisher Scientific) to achieve a final concentration of 1.5 mM and incubated for 25 min in darkness at room temperature before flow cytometry (FCM) analysis. The FCM analysis was performed with a BD Accuri C6 flow cytometer. The 5-CFDA (second tube) was excited by the flow cytometer laser at 488 nm and emitted green fluorescence collected by the filter 530 nm + /− 15 nm (FL1 channel). The results were compared to the control tube to eliminate cellular autofluorescence. Data were analyzed using statistic tables that indicate the number and percentage of viable cells as well as the fluorescence intensity.

### Fermentations

Fermentations were carried out in duplicate in white must (Chardonnay) containing 212 g.L^−1^ of glucose/fructose, a pH of 3.41 and 251 mg.L^−1^ of total assimilable nitrogen. The must was centrifuged at 7,000 g for 7 min at 4 °C. Sugar concentration and ethanol production were monitored by Fourier transform infrared spectroscopy (FTIR, OenoFOSS™, FOSS, Hilleroed, Denmark).

Single-culture fermentations were carried out in 250 mL Erlenmeyer flasks, closed with sterile cotton wool, containing 100 mL of Chardonnay must. Each sample was inoculated with pre-cultured yeast cells (10^6^ cells.mL^−1^) and incubated at 20 °C without agitation.

Sequential fermentations were carried out in 250 mL Erlenmeyer flasks, filled with 100 mL of must and inoculated with 10^6^ cells.mL^−1^ non*-Saccharomyces* (NS) yeast (SB1, SB2, LT or Mp). 24 h after the NS yeast inoculation, a second inoculation with 10^6^ cells.mL^−1^ *Saccharomyces cerevisiae* was done.

### Direct infusion FT-ICR-MS

Ultrahigh-resolution FT-ICR-MS were acquired with a 12 T Bruker Solarix mass spectrometer (Bruker Daltonics, Bremen, Germany) equipped with an APOLLO II electrospray source in negative ionization mode^48^. For MS analysis, samples were diluted 1:100 (*v/v*) in methanol (LC-MS grade, Fluka, Germany)^7,49^. The diluted samples were infused into the electrospray ion source with a flow rate of 120 μL.h^−1 ^^48,49^. Settings for the ion source were: drying gas temperature 180 °C, drying gas flow 4.0 L.min^−1^, capillary voltage 3,600 V. Spectra were first externally calibrated by ion clusters of arginine (10 ppm in methanol). Internal calibration of each spectrum was conducted with a reference list including selected wine makers and ubiquitous fatty acids. The spectra were acquired with a time-domain of 4 megawords and 400 scans were accumulated within a mass range of *m/z* 92 to 1000. A resolving power of 400,000 at *m/z* 300 was achieved^48,49^.

### Processing of FT-ICR-MS data

Raw spectra were post-processed by Compass DataAnalysis 4.2 (Bruker Daltonics, Bremen, Germany) and peaks with a signal-to-noise ratio (S/N) of at least 6 were exported to mass lists^49^. All exported *m/z* features were aligned into a matrix containing averaged *m/z* values (peak alignment window width: ±1 ppm) and corresponding peak intensities of all analyzed samples^48^. Molecular formulae were assigned to the exact *m/z* values by mass difference network analysis using an in-house developed software tool^60^. In total, the matrix containing the entire sample set contained 5979 detected features that could be assigned to distinct and unique molecular formulae. More than 90% of all assignments were found within an error range lower than 0.2 ppm. All further calculations and filtering were done in Perseus 1.5.1.6 (Max Planck Institute of Biochemistry, Germany) and R Statistical Language (version 3.1.1).

### Repeatability of FT-ICR-MS measurements

Quality control (QC) samples were prepared by pooling equal amounts of all samples. QC samples were injected at the beginning and after every 10 samples to monitor the reproducibility of the measurements overtime (Supplementary Fig. 7). The repeatability over the entire time of analysis was evaluated by calculating the coefficient of variation (CV) from the peak intensities of all elemental compositions detected in the QC samples. More than 95% of all elemental compositions showed a CV-value lower than 20% (Supplementary Fig. 7C). All samples were prepared in duplicates. Each biological replicate was analyzed in duplicate (N = 2×2).

### UHPLC-QToF-MS/MS experiments

Discriminative markers found after chemometric analyses were subjected to tandem-MS experiments. Samples were directly injected into the LC-MS/MS system without dilution. Metabolites were separated using a Waters Acquity UPLC system coupled to a Bruker maXis UHR-ToF-MS. A reversed-phase (RP) separation method was employed which separates middle to non-polar metabolites using a BEH C18 column (100 × 2.1 mm ID, 1.7 μm; (Waters, City, Country)^49^. The column temperature was set to 40 °C. Eluent A consisted of 10% acetonitrile (ACN) in water and Eluent B of 100% ACN, both with 0.1% formic acid^49^. Detection was carried out in negative ionization mode with the following parameters: Nebulizer pressure = 2.0 bar, dry gas flow = 8.0 l.min^−1^, dry gas temperature = 200 °C, capillary voltage = 3,500 V, end plate offset = −500 V^49^. The flow rate was 0.4 mL.min^−1^. UHR-ToF-MS acquisitions were carried out in profile spectra mode with a total scanning rate of 1 Hz. Instrument tuning focused on detection and resolution of molecular weight compounds in the mass range of 50 to 2,000 Da. Mass calibration was carried out with low concentration ESI Tuning Mix (Agilent, Waldbronn, Germany)^49^. After acquisition, MS/MS spectra were manually extracted using Bruker Data Analysis 4.4 (Bruker Daltonic, Bremen, Germany).

### Sample preparation

Samples prepared by direct dilution with methanol (1:100 *v/v*) and after purification using C18 SPE cartridges (100 mg.ml^−1^ Backerbond SPE columns) were compared by FT-ICR-MS (Supplementary Figs. 8 and 9). The cartridges were used with pH = 2 and methanol elution. Principal component analysis computed from all analyzed samples (direct dilution and after SPE purification) clearly distinguished the two sample preparation methods (Supplementary Fig. 8). About 50% of the detected molecular compositions were recovered after both sample preparation protocols (Supplementary Fig. 9). The number and the nature of compounds specific to the sample preparation was 1259 and 1052 molecular formulae detected in the samples after SPE extraction and after direct dilution, respectively (Supplementary Fig. 9). The molecular composition after SPE extraction was mainly comprised of CHO and CHNO compounds whereas the molecular composition after direct dilution was more equally distributed between CHO, CHOS, CHNO and CHNOS (Supplementary Figs. 8 and 9). We found possible hits in databases for 228 and 164 of the observed molecular formulae after direct dilution and SPE purification, respectively (Supplemental Fig. 9). Corresponding metabolic pathways are shown in Supplementary Fig. 10. High numbers of compounds were annotated in pathways representing metabolism of carbohydrates, amino acids and polyphenols. Samples analyzed after direct dilution showed stronger selectivity for carbohydrate-type compounds while polyphenols showed good enrichment on the C18 material. Based on the FT-ICR-MS results, both preparation methods are interesting, however, because of the faster sample preparation and easier combination with LC-MS data, methanol dilution was used as preferred sample preparation method throughout the following study.

### Statistical analysis

All samples were prepared in duplicates. Each biological replicate was analyzed in duplicate (N = 2×2). Principal component analysis (PCA), hierarchical cluster analysis (HCA) and analysis of variance (ANOVA) were performed using Perseus 1.5.1.6 (Max Planck Institute of Biochemistry, Germany). For HCA, Euclidean distance and average linkage were chosen. Van Krevelen diagrams (O/C versus H/C elemental ratios) and multidimensional stoichiometric compounds classification (MSCC) have been used to elucidate main compound categories commonly defined as lipids, peptides, amino sugars, carbohydrates, nucleotides and polyphenols compounds^47,48^.

## Supplementary information

Supplementary information.

## Data Availability

The datasets generated or analyzed during this study are included in this published article and its Supplementary Information files.
