# Supplementary material for: Exploring yeast interactions through metabolic profiling
Source: Sci Rep. 2020 Apr 8;10:6073. doi: 10.1038/s41598-020-63182-6 (PMC7142100; doi:10.1038/s41598-020-63182-6)
Supplement: Supplementary file 1 — Supplementary information. [file 41598_2020_63182_MOESM1_ESM.docx]

Exploring yeast interactions through metabolic profiling

C Roullier-Gall, V David, D Hemmler, P Schmitt-Kopplin and H Alexandre

**Supporting information**

Table of contents

[Supplemental figure 1 2](#_Toc20230517)

[Supplemental figure 2 3](#_Toc20230518)

[Supplemental figure 3 4](#_Toc20230519)

[Supplemental figure 4 5](#_Toc20230520)

[Supplemental figure 5 6](#_Toc20230521)

[Supplemental figure 6 7](#_Toc20230522)

[Supplemental figure 7 7](#_Toc20230523)

[Supplemental figure 8 8](#_Toc20230524)

[Supplemental figure 9 8](#_Toc20230525)

[Supplemental table 1 9](#_Toc20230526)

[Supplemental table 2 14](#_Toc20230527)


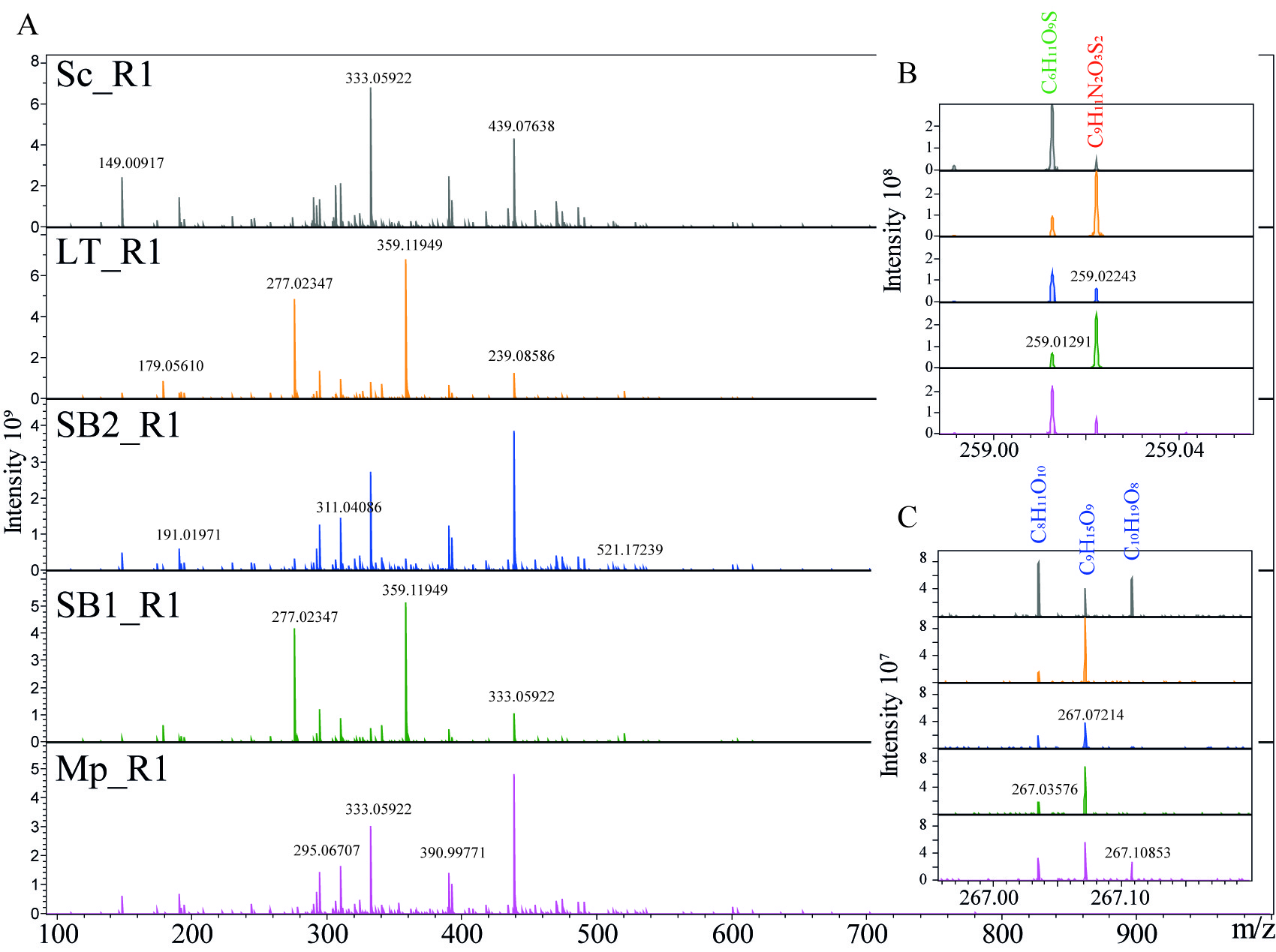


Supplemental figure 1: Visualization of ESI(-) FT-ICR-MS spectra of five wine samples which only differ in the yeast strain used for alcoholic fermentation (mass range: 100–1,000 Da). Enlargements of the nominal masses at *m/z* 259 and *m/z* 267 illustrate the molecular formula assignments.


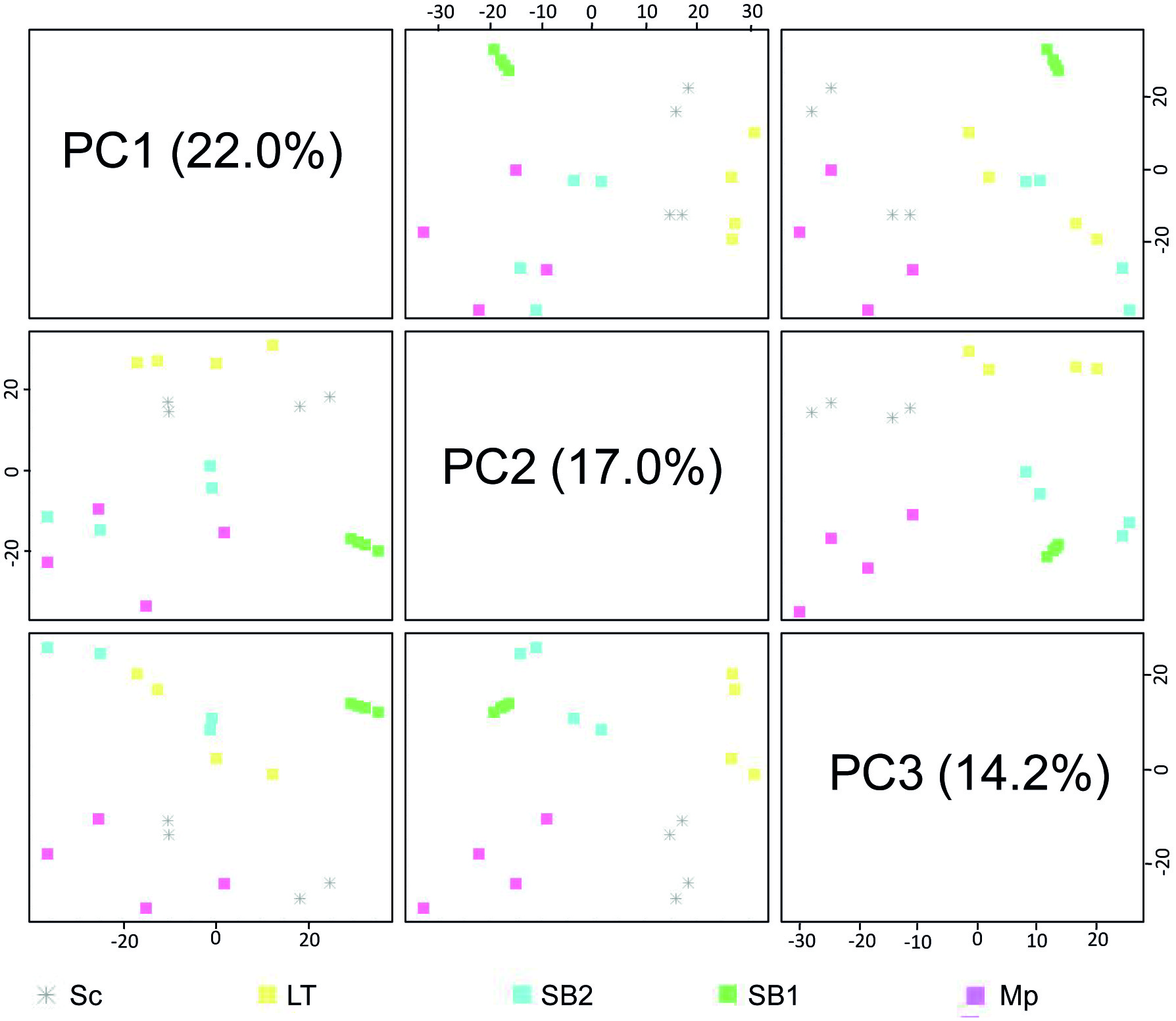


Supplemental figure 2: Scores plots of the first three principal components computed from the samples fermented with Sc, LT, SB1, SB2 and Mp. The three first components explained 53.2% of the total variability.


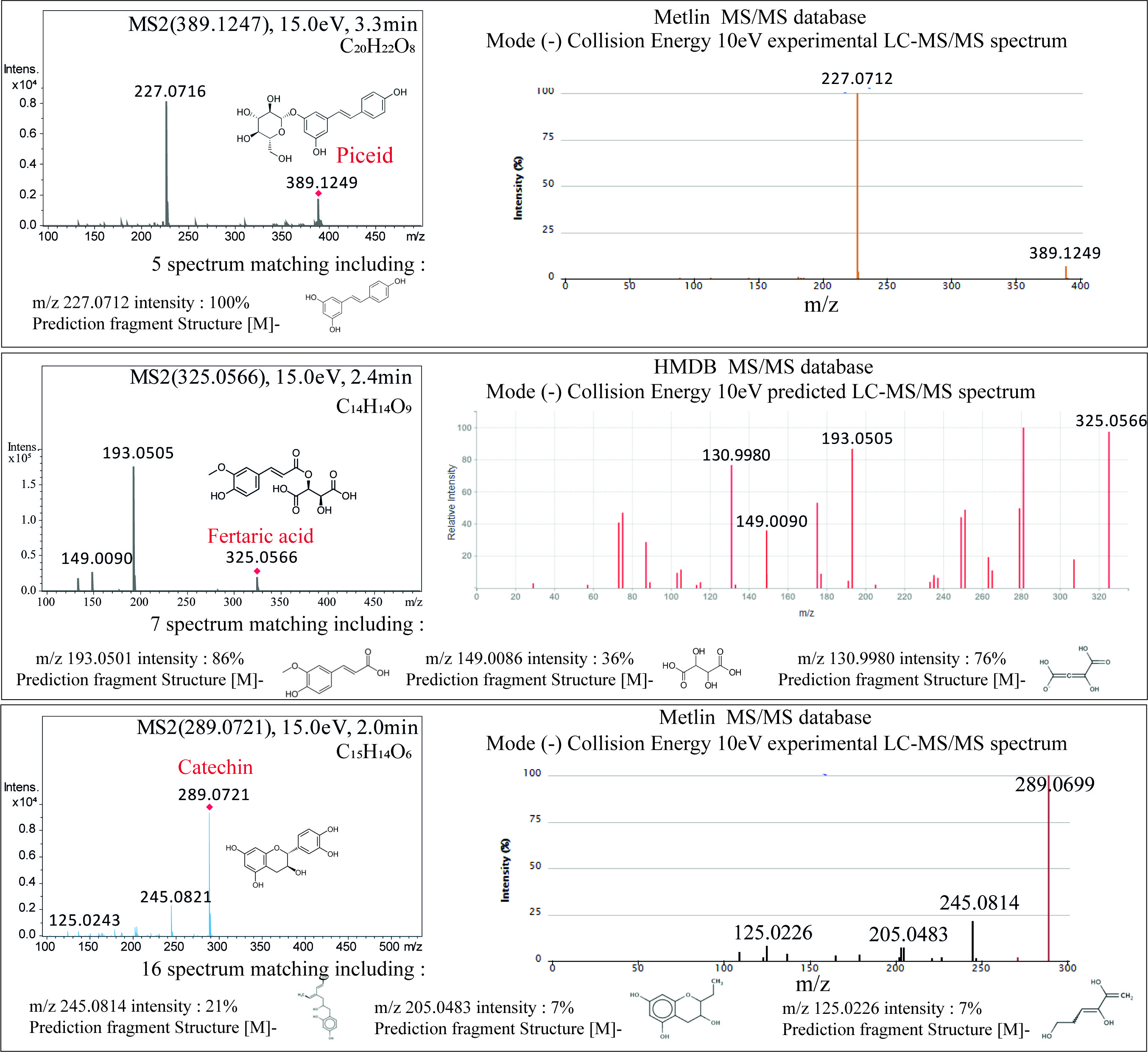


Supplemental figure 3**:** Piceid, Fertaric acid and catechin comparison of MS/MS spectra (15eV) from wine samples and MS/MS databases.


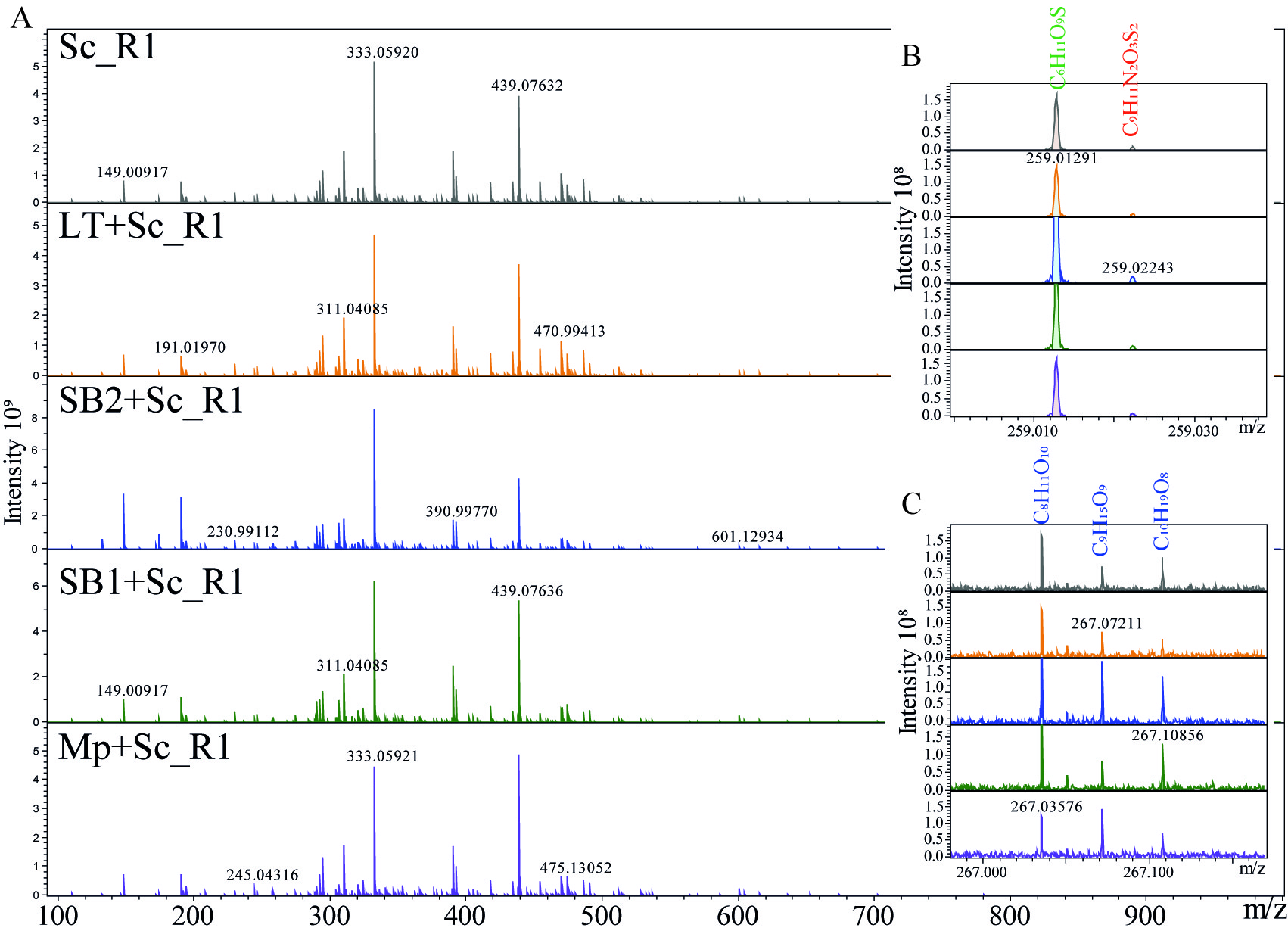


Supplemental figure 4: Visualization of ESI(-) FT-ICR-MS spectra of five wine samples which only differ by the yeast used for alcoholic fermentation (Sc alone, LT+Sc, SB2+Sc, SB1+Sc and Mp+Sc), in the mass range from 100–1,000 Da together with enlargements of the nominal masses *m/z* 259 and *m/z* 267 showing the molecular formula assignments.


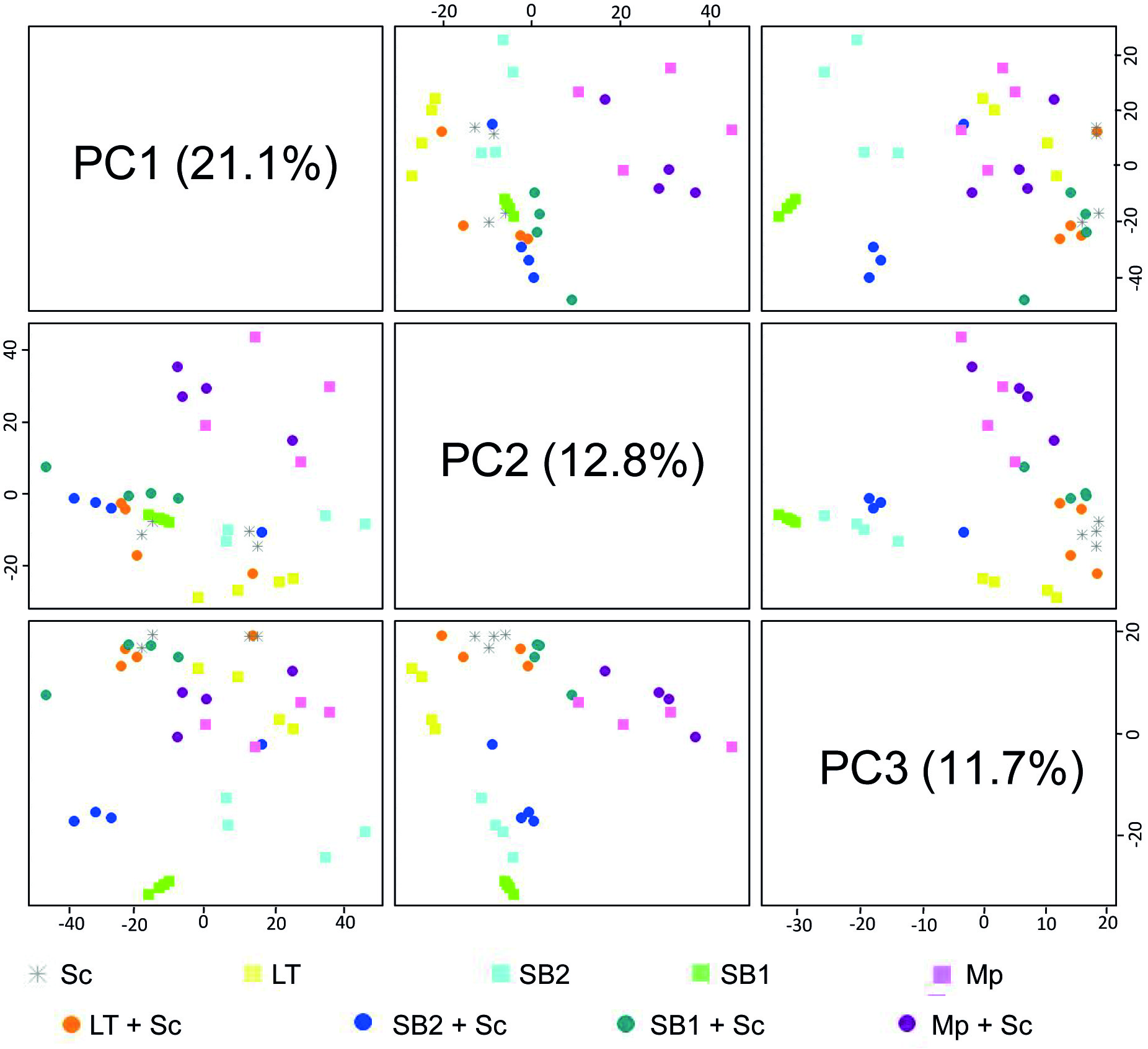


Supplemental figure 5: Scores plots of the first three principal component analysis (PCA) of the samples from Sc, LT, SB1, SB2, Mp, LT+Sc, SB1+Sc, SB2+Sc and Mp+Sc fermentations using direct methanol dilution. The three first components explained 45.6% of the total variability.

**
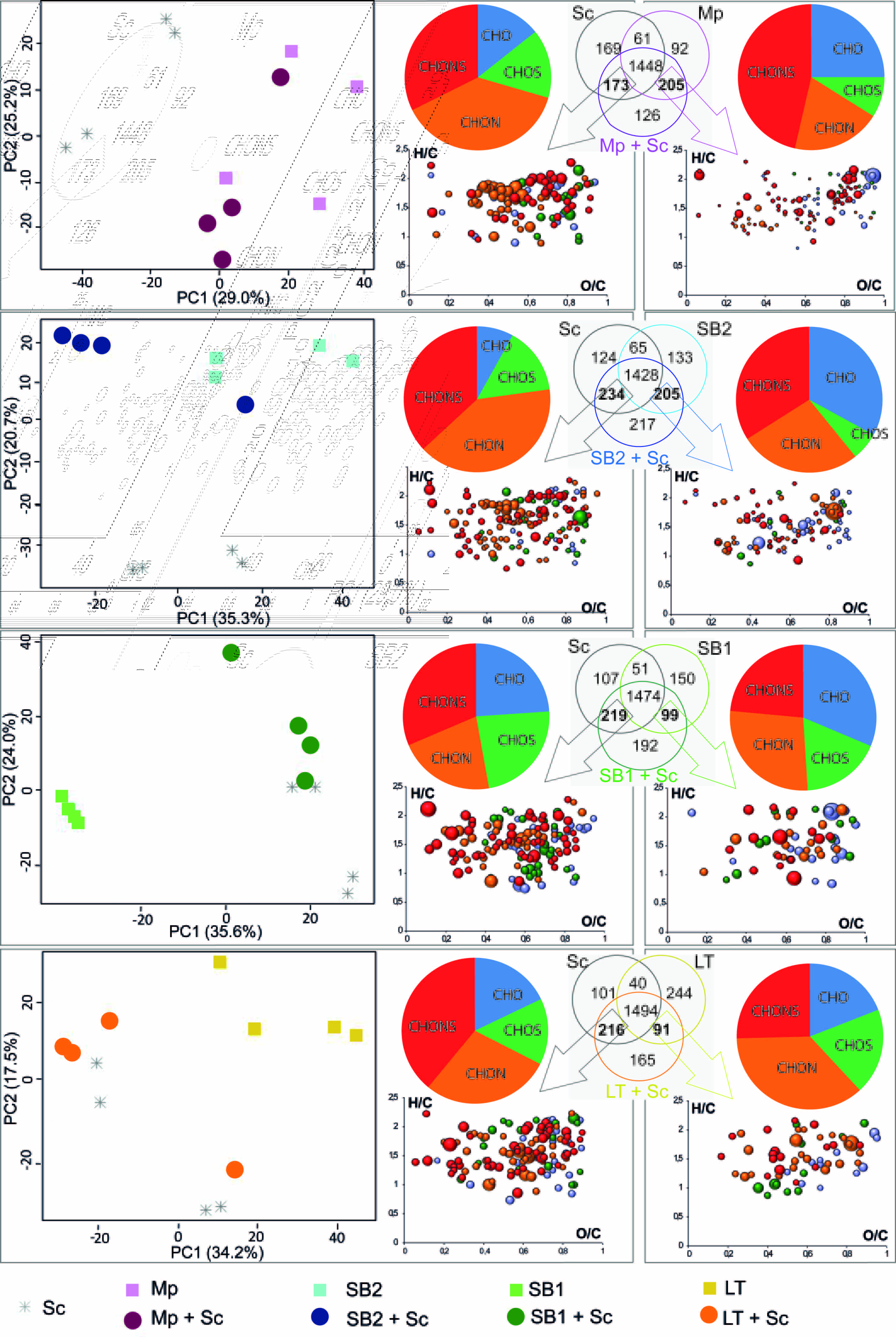
**

Supplemental figure 6 : Principal component analysis of single fermentations and mixed fermentation with Venn diagrams for each NS yeast. Markers of Sc and each NS yeast in sequential fermentation are highlight in the van Krevelen diagrams. Bubble sizes indicate relative intensities of corresponding peaks in the spectra. Color code: CHO, blue; CHOS, green; CHON, red; CHONS, orange.


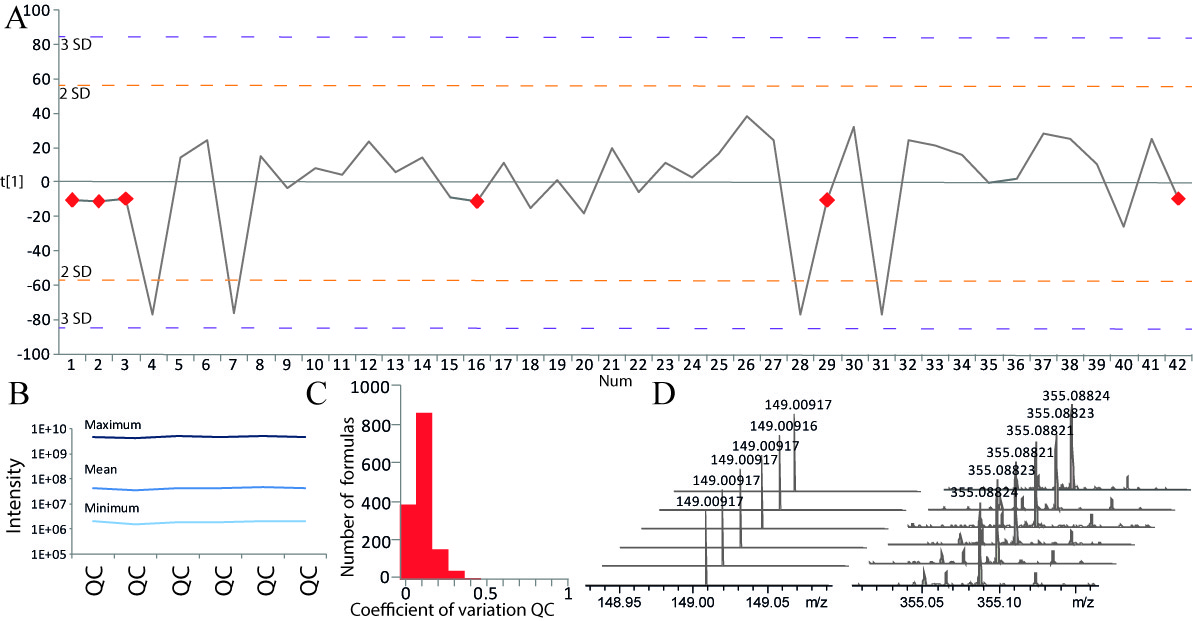


Supplemental figure 7: (A) Time series plot of the first principal component (t(1) vs. sample run order). QCs are highlighted as red diamonds. (B) Plot representing the maximum, the mean and the minimum of peak intensity for each of the 6 QCs. (C) Histogram of all coefficient of variation vaues computed from the peak intensities from all dectected molecular compositions in the FT-ICR-MS data. (D) Extracted ion chromatograms of *m/z* 149.00917 and 355.08821 in 6 sequential QCs samples.


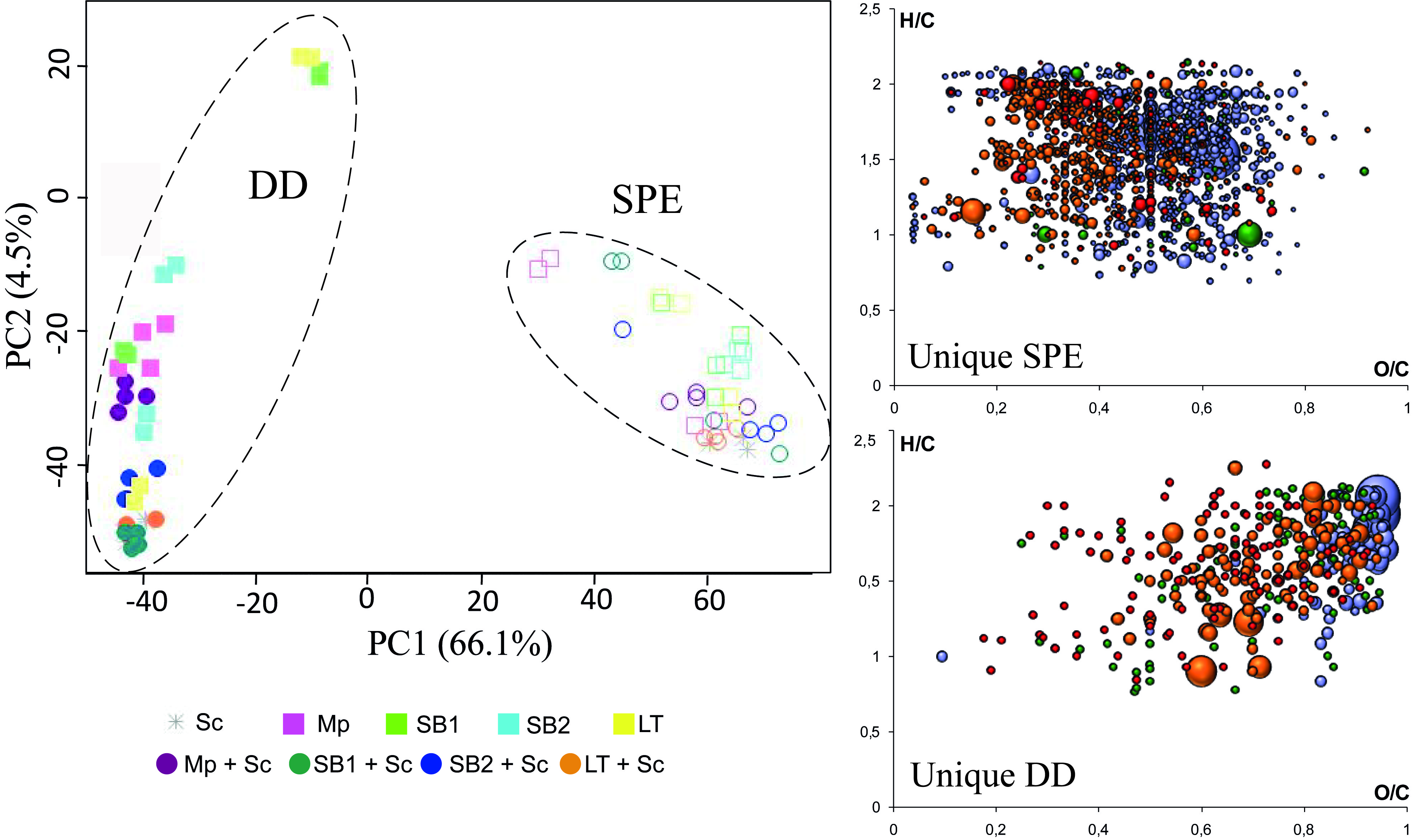


Supplemental figure 8: Comparison of samples depending on the sample preparation : direct methanol dilution with methanol (DD, open symbols) and purification by C18 SPE c (C18, solid symbols). Van Krevelen diagrams show molecular formulae only found after methanol dilution (DD) or C18 purification (C18).


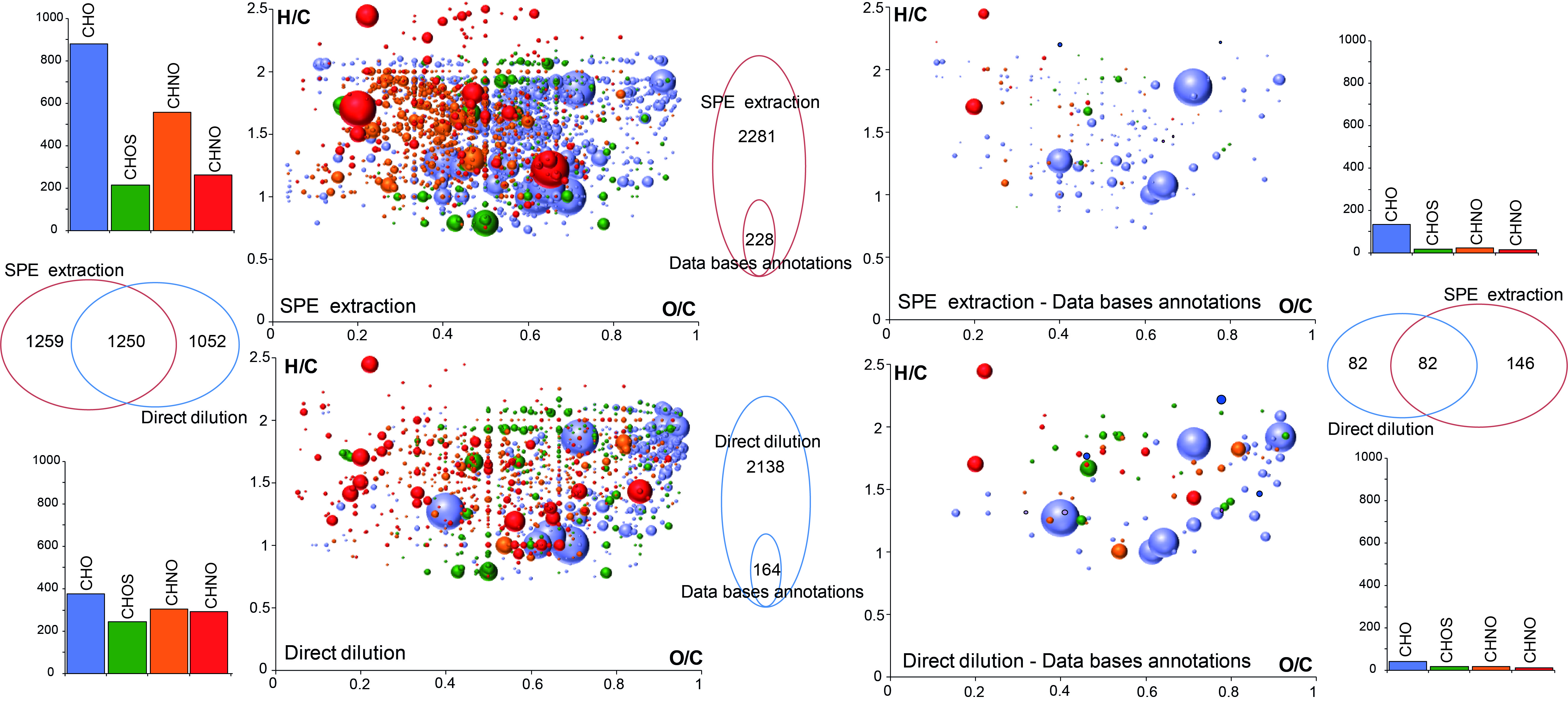


Supplemental figure 9: Common and unique compounds detected after methanol dilution (Direct Dilution) or C18 purification (SPE extraction).


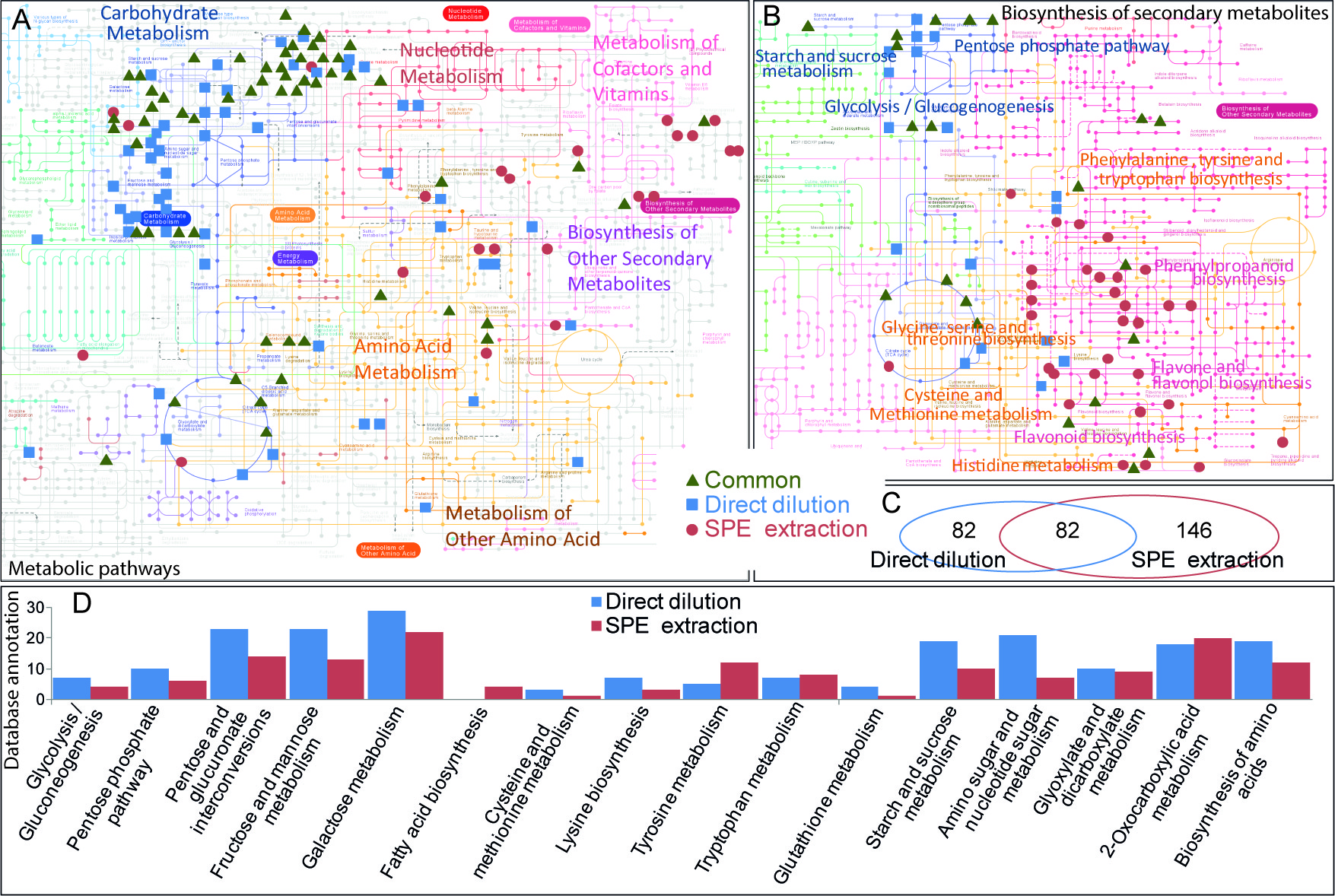


Supplemental figure 10: KEGG representation showing an overview of (A) metabolic pathways and a zooming into (B) biosynthesis pathways of secondary metabolites. Metabolites identified in the samples after direct dilution are indicated as blue squares and after SPE purification as red circles. (C) Venn diagrams of the number of annotations in databases using direct dilution and SPE.(D) Number of database annotations in 16 pathways found for the direct dilution (blue) and SPE extraction (red) method.

Supplemental table 1**:** Database annotations of markers from SB1, SB2, LT, Mp and Sc fermentations

| **Mass (avg.)** | **Yeast** | **Formulas [M]** | **Database annotations** |
| --- | --- | --- | --- |
| 103.003694 | SB1 | C3H4O4 | Malonate |
| 111.008782 | SB1 | C5H4O3 | 2-Furoic acid |
| 115.00369 | SB1 | C4H4O4 | 2-Butenedioic acid (2E)-; (Fumaric acid) |
| 124.99141 | SB1 | C2H6O4S | 2-Hydroxyethanesulfonate |
| 125.024425 | SB1 | C6H6O3 | Pyrogallol (8CI); 1,2,3-Benzenetriol; 1,3,5-Benzenetriol; 2-Furancarboxaldehyde, (hydroxymethyl)-; 2-Furancarboxaldehyde, 5-(hydroxymethyl)-; 4H-Pyran-4-one, 3-hydroxy-2-methyl- ; phloroglucinol; 1,2,3-Trihydroxybenzene |
| 129.019338 | SB1 | C5H6O4 | Glutaconic acid |
| 133.014253 | SB1 | C4H6O5 | Malic acid;L-(-)-Malic acid |
| 133.050637 | SB1 | C5H10O4 | 2,3-Dihydroxyvaleric acid |
| 143.034988 | SB1 | C6H8O4 | 4H-Pyran-4-one, 2,3-dihydro-3,5-dihydroxy-6-methyl-;3-Hexenedioic acid |
| 145.01425 | Mp | C5H6O5 | Glutaric acid, 2-oxo- (8CI);alpha-Ketoglutaric acid |
| 147.0299 | SB1 | C5H8O5 | Citramalic acid |
| 149.009167 | SB1 | C4H6O6 | L-(+)-Tartaric acid; Butanedioic acid, 2,3-dihydroxy-; Tartaric acid |
| 149.045551 | SB1 | C5H10O5 | D-Xylose; D-Ribose; Xylose; Arabinose; L-Arabinose |
| 151.061198 | Mp | C5H12O5 | Xylitol;Arabinitol |
| 154.998599 | SB1 | C6H4O5 | 2,5-Furandicarboxylic acid |
| 157.050636 | SB1 | C7H10O4 | 3-Furancarboxylic acid, tetrahydro-5-oxo-, ethyl ester; 2-Furancarboxylic acid, tetrahydro-5-oxo-, ethyl ester; Succinylacetone |
| 159.0299 | SB1 | C6H8O5 | Oxoadipic acid |
| 161.045549 | SB1 | C6H10O5 | Dicarbonic acid, diethyl ester (9CI);1,6-Anhydroglucose |
| 163.040068 | SB1 | C9H8O3 | 2-Propenoic acid, 3-(hydroxyphenyl)-,3-(4-hydroxyphenyl)-; trans-p-Coumaric acid; 4-Coumarate |
| 165.040463 | SB1 | C5H10O6 | Arabinonic acid |
| 169.014247 | SB1 | C7H6O5 | Gallate;Benzoic acid, 3,4,5-trihydroxy- |
| 171.066282 | SB2 | C8H12O4 | 2-Octenedioic acid |
| 173.00916 | SB1 | C6H6O6 | cis-Aconitic acid |
| 175.024809 | SB1 | C6H8O6 | L-Ascorbic acid (8CI,9CI); erythorbic acid |
| 175.061195 | SB1 | C7H12O5 | 2-Isopropylmalic acid |
| 177.04046 | SB1 | C6H10O6 | D-Gluconic acid, d-lactone (9CI); Fructose, 5-dehydro-, D- (6CI); L-Galactono-1,4-lactone |
| 179.034979 | SB1 | C9H8O4 | caffeic acid; 2-Propenoic acid, 3-(3,4-dihydroxyphenyl)-; Caffeate |
| 179.03835 | SB1 | C6H12O4S | 5-Methylthioribose |
| 179.056109 | SB1 | C6H12O6 | D-Glucose; D-Mannose; D-Fructose; D-Galactose; myo-Inositol |
| 181.05063 | SB2 | C9H10O4 | 2',6'-Dihydroxy-4'-methoxyacetophenone; Hydrocinnamic acid, 3,4-dihydroxy- (6CI,7CI,8CI); Syringaldehyde; Benzaldehyde, 4-hydroxy-3,5-dimethoxy-; Methyl vanillate |
| 181.071759 | Mp | C6H14O6 | D-Glucitol , Sorbitol; Galactitol; Mannitol ; D-Mannitol |
| 187.024806 | SB1 | C7H8O6 | cis-2-Methylaconitate |
| 188.05644 | SB1 | C7H11NO5 | Glutarylglycine |
| 189.004069 | SB1 | C6H6O7 | Oxalosuccinic acid |
| 189.040455 | SB1 | C7H10O6 | 4-Hydroxy-2-oxo-heptandioic acid |
| 189.07684 | SB1 | C8H14O5 | Butanedioic acid, hydroxy-, diethyl ester; 3-Hydroxysuberic acid; Diethyl malate |
| 191.019719 | SB1 | C6H8O7 | 1,2,3-Propanetricarboxylic acid, 2-hydroxy-; citric acid; Isocitric acid; 2,5-Diketo-D-gluconic acid |
| 191.056105 | SB1 | C7H12O6 | Quinic acid |
| 191.107742 | SB1 | C12H16O2 | Thymyl acetate;Propanoic acid, 2-methyl-, 2-phenylethyl ester |
| 193.035369 | SB1 | C6H10O7 | D-arabino-5-Hexulosonic acid (7CI,8CI,9CI);D-arabino-Hexonic acid, 2-keto- (6CI); D-Galacturonic acid;D-Galacturonic acid, homopolymer (9CI); 5-Oxo-D-gluconic acid; D-glucuronate |
| 193.050625 | SB1 | C10H10O4 | Ferulic acid; 2-Propenoic acid, 3-(4-hydroxy-3-methoxyphenyl)-; Ferulate; 2-Propenoic acid, 3-(4-hydroxy-3-methoxyphenyl)-; Ferulic acid; Vanilloylacetyl; 1,2-Propanedione, 1-(4-hydroxy-3-methoxyphenyl)-; Vanilloyl methyl ketone |
| 195.051019 | SB1 | C6H12O7 | D-Gluconic acid ;D-Gluconate |
| 197.045539 | SB1 | C9H10O5 | Syringic acid (6CI); Benzoic acid, 4-hydroxy-3,5-dimethoxy-;Ethyl gallate |
| 203.019718 | SB1 | C7H8O7 | 2-Oxaloglutaric acid |
| 205.035368 | SB1 | C7H10O7 | 2-Methylcitric acid |
| 206.045873 | SB1 | C10H9NO4 | 4-(2-Aminophenyl)-2,4-dioxobutanoic acid |
| 211.024802 | SB1 | C9H8O6 | 5-carboxyvanillic acid |
| 213.017048 | SB1 | C5H11O7P | D-threo-2-Pentulose, 1-deoxy-, 5-(dihydrogen phosphate) (9CI);1-Deoxy-D-xylulose 5-phosphate |
| 214.048588 | Mp | C5H14NO6P | Glycerylphosphorylethanolamine |
| 215.032678 | SB1 | C5H13O7P | 1,2,3,4-Butanetetrol, 2-methyl-, 4-(dihydrogen phosphate), (2S,3R)- (9CI); 2-C-Methyl-D-erythritol 4-phosphate; Methylerythritol phosphate |
| 216.981226 | SB1 | C7H6O6S | 5-Sulfosalicylic acid |
| 218.066992 | SB1 | C8H13NO6 | O-Succinyl-L-homoserine |
| 243.062252 | SB1 | C9H12N2O6 | Uridine (8CI,9CI) |
| 253.092888 | SB1 | C9H18O8 | Galactosylglycerol |
| 255.232949 | SB1 | C16H32O2 | Tetradecanoic acid, ethyl ester; Palmitic acid; Hexadecanoic acid; Hexadecanoic acid |
| 257.077908 | SB1 | C10H14N2O6 | Ribothymidine |
| 258.038425 | Mp | C6H14NO8P | Glucosamine-1P |
| 261.007446 | Sc | C9H10O7S | Homovanillic acid sulfate |
| 261.038089 | Mp | C6H15O9P | Mannitol 1-phosphate |
| 261.072819 | SB1 | C9H14N2O7 | L-glutamic acid, L-a-aspartyl;L-beta-aspartyl-L-glutamic acid |
| 262.039076 | LT | C9H13NO6S | Epinephrine sulfate |
| 267.072151 | SB1 | C9H16O9 | 3-Deoxy-D-glycero-D-galacto-2-nonulosonic acid |
| 274.104454 | SB1 | C10H17N3O6 | Norophthalmic acid |
| 275.088476 | SB1 | C10H16N2O7 | L-glutamic acid, L-a-glutamyl;Gamma Glutamylglutamic acid |
| 275.092502 | SB1 | C15H16O5 | Dihydromethysticin |
| 275.124856 | Sc | C11H20N2O6 | Saccharopine |
| 277.122749 | SB2 | C11H22N2O4S | Pantetheine |
| 284.125185 | SB1 | C12H19N3O5 | Glycylprolylhydroxyproline |
| 288.120109 | SB1 | C11H19N3O6 | Ophthalmic acid |
| 289.033011 | SB1 | C7H15O10P | D-Sedoheptulose 7-phosphate |
| 289.071762 | SB1 | C15H14O6 | (-)-Epicatechin; 2H-1-Benzopyran-3,5,7-triol, 2-(3,4-dihydroxyphenyl)-3,4-dihydro-, (2R,3R)-; (+)-Catechin; 2H-1-Benzopyran-3,5,7-triol, 2-(3,4-dihydroxyphenyl)-3,4-dihydro-, (2R,3S)- |
| 289.115348 | LT s | C10H18N4O6 | Argininosuccinic acid |
| 295.04594 | SB1 | C13H12O8 | trans-Coumaroyltartaric acid; trans-Coutaric acid; Butanedioic acid, 2-hydroxy-3-[[(2E)-3-(4-hydroxyphenyl)-1-oxo-2-propenyl]oxy]-, (2R,3R)-; 2-hydroxy-3-[[3-(4-hydroxyphenyl)-1-oxo-2-propenyl]oxy]-, (2R,3R)-; cis-Coumaroyltartaric acid; cis-Coutaric acid; Butanedioic acid, 2-hydroxy-3-[[(2Z)-3-(4-hydroxyphenyl)-1-oxo-2-propenyl]oxy]-, (2R,3R)- |
| 299.077244 | SB1 | C13H16O8 | Pseudolaroside A |
| 300.048993 | Mp | C8H16NO9P | N-Acetyl-D-Glucosamine 6-Phosphate |
| 302.135758 | Sc | C12H21N3O6 | nicotianamine |
| 303.083375 | SB1 | C11H16N2O8 | N-Acetylaspartylglutamic acid |
| 305.018007 | Sc | C9H11N2O8P | Uridine 2',3'-cyclic phosphate |
| 306.07653 | Sc | C10H17N3O6S | L-Glutathione; Glycine, L-g-glutamyl-L-cysteinyl- |
| 308.09871 | SB1 | C11H19NO9 | N-Acetylneuraminic acid |
| 311.040858 | SB1 | C13H12O9 | trans-Caftaric acid; Caftaric acid; Monocaffeoyl tartrate; Caftaric acid;Butanedioic acid, 2-[[(2E)-3-(3,4-dihydroxyphenyl)-1-oxo-2-propenyl]oxy]-3-hydroxy-, (2R,3R)-; Butanedioic acid, 2-[[(2Z)-3-(3,4-dihydroxyphenyl)-1-oxo-2-propenyl]oxy]-3-hydroxy-, (2R,3R)- |
| 311.098374 | Mp | C11H20O10 | Galactose-beta-1,4-xylose |
| 311.11363 | SB1 | C15H20O7 | 4-Hydroxycinnamyl alcohol 4-D-glucoside |
| 313.056507 | SB1 | C13H14O9 | 1-Salicylate glucuronide |
| 315.072158 | SB2 | C13H16O9 | Protocatechuic acid 4-O-glucoside |
| 315.108545 | SB1 | C14H20O8 | Hydroxytyrosol 4-O-glucoside |
| 317.027935 | SB1 | C8H15O11P | 3-deoxy-D-manno-octulosonate 8-phosphat |
| 321.005311 | Sc | C14H6O8 | ellagic acid |
| 322.044574 | Sc | C9H14N3O8P | Cytidine monophosphate;3'-Cytidylic acid (8CI,9CI) |
| 323.028595 | Sc | C9H13N2O9P | UMP;2'-Uridylic acid (8CI,9CI) |
| 325.056511 | SB1 | C14H14O9 | t-Feruloyl tartaric acid; trans-Fertaric acid;Monoferuloyl tartrate |
| 325.092896 | SB2 | C15H18O8 | (Z)-; 4-O-b-D-Glucopyranosyl-cis-p-coumaric acid; trans-beta-D-Glucosyl-2-hydroxycinnamate; p-Hydroxy-trans-cinnamic acid b-D-glucosyl ester; (E)-; 4-O-b-D-Glucopyranosyl-p-trans-coumaric acid |
| 325.11403 | Mp | C12H22O10 | 2-O-a-L-Fucopyranosyl-galactose |
| 327.108543 | SB1 | C15H20O8 | Anisatin |
| 329.087812 | SB2 | C14H18O9 | Pseudolaroside B;D-Glucose 1-vanillate; Vanillic acid 1-O-b-D-glucopyranosyl ester |
| 331.067076 | SB2 | C13H16O10 | gallate + glucose;b-D-Glucose, 1-gallate (6CI) |
| 333.059226 | LT | C9H19O11P | 1-(sn-Glycero-3-phospho)-1D-myo-inositol |
| 339.072157 | LT | C15H16O9 | Sinapoyl malate; Butanedioic acid, 2-[[(2E)-3-(3,4-dihydroxyphenyl)-1-oxo-2-propenyl]oxy]-3-hydroxy-, 1-ethyl ester, (2R,3R)- (9CI); , 4-ethyl ester, (2R,3R)- (9CI); 2H-1-Benzopyran-2-one, 6-(b-D-glucopyranosyloxy)-7-hydroxy- |
| 339.093292 | SB1 | C12H20O11 | 3'-Ketolactose |
| 341.087815 | SB2 | C15H18O9 | Caffeic acid 3-glucoside |
| 341.108943 | Mp | C12H22O11 | Maltose; D-Glucopyranose, 4-6-O-a-D-galactopyranosyl-; D-Glucose, 4-O-a-D-glucopyranosyl-,D-Glucose, 4-O-b-D-galactopyranosyl-,6-O-a-D-galactopyranosyl-; D-melibiose; Trehalose (8CI); a-D-Glucopyranoside, a-D-glucopyranosyl; a-D-Glucopyranoside, b-D-fructofuranosyl |
| 343.12459 | Mp | C12H24O11 | Maltitol |
| 344.040168 | Sc | C10H12N5O7P | Cyclic GMP |
| 345.119114 | SB1 | C15H22O9 | Aucubin |
| 346.055818 | Sc | C10H14N5O7P | Adenosine phosphate; 3'-Adenylic acid (8CI,9CI); Adenosine monophosphate |
| 347.038554 | Sc | C9H17O12P | 2-O-(6-Phospho-alpha-mannosyl)-D-glycerate |
| 357.119121 | SB1 | C16H22O9 | 10-Deoxygeniposidic acid |
| 359.098382 | SB2 | C15H20O10 | 3-Methoxy-4-hydroxyphenylglycol glucuronide |
| 359.134766 | SB1 | C16H24O9 | 7-Deoxyloganate |
| 361.114027 | SB2 | C15H22O10 | Antirrhinoside |
| 362.050727 | Sc | C10H14N5O8P | 3'-2'-Guanylic acid (8CI,9CI); Guanosine monophosphate |
| 366.140542 | SB1 | C14H25NO10 | N-Acetyl-6-O-L-fucosyl-D-glucosamine |
| 367.018473 | Mp | C10H13N2O11P | Orotidine 5'-phosphate |
| 369.140246 | Mp | C14H26O11 | Amylose |
| 373.114039 | SB2 | C16H22O10 | Geniposidic acid |
| 377.130072 | Mp | C12H26O13 | Galactinol dihydrate |
| 382.135505 | SB1 | C14H25NO11 | N-Acetyllactosamine |
| 387.166078 | SB1 | C18H28O9 | Tuberonic acidglucoside |
| 389.10897 | SB2 | C16H22O11 | Monotropein |
| 389.124212 | SB2 | C20H22O8 | Resveratrol 3-O-b-glucopyranoside; trans-Piceid; cis-Piceid; D-Glucitol, 1,5-anhydro-1-C-[2,4-dihydroxy-6-[(1E/ Z)-2-(4-hydroxyphenyl)ethenyl]phenyl]-, (1S)- (9CI) |
| 414.118606 | Sc | C15H22N5O7P | Isopentenyl adenosine monophosphate |
| 421.075299 | Mp | C12H23O14P | alpha,alpha-Trehalose 6-phosphate |
| 430.11354 | Sc | C15H22N5O8P | cis-zeatin riboside monophosphate |
| 439.106851 | Sc | C20H24O9S | Hallactone B |
| 444.129187 | Sc | C16H24N5O8P | O-Hexanoyl-adnosine monophosphate |
| 449.108977 | SB2 | C21H22O11 | 2',3,4,4',6'-Peptahydroxychalcone 4'-O-glucoside; 2R,3R-Dihydrokaempferol 3-O-b-D-glucoside; Arthromerin B; Astilbin (6CI,7CI,8CI); Taxifolin 3-O-rhamnoside |
| 464.096244 | SB1 | C21H21O12 | Delphinidin 3-O-glucoside |
| 480.981928 | LT | C10H17N2O14P3 | Thymidine 5'-triphosphate |
| 487.166901 | Sc | C18H32O15 | 3-Fucosyllactose |
| 503.161807 | LT | C18H32O16 | Raffinose;a-D-Glucopyranoside, b-D-fructofuranosyl O-a-D-galactopyranosyl-(1½6)- |
| 535.15165 | Mp | C18H32O18 | 1,4-beta-D-Glucan |
| 551.177065 | LT | C26H32O13 | cis-Resveratrol-3,4'-O-beta-diglucoside |
| 599.104352 | LT | C28H24O15 | Kaempferol 3-(6''-galloylgalactoside) |
| 616.109115 | SB1 | C23H27N3O15S | Glycine, L-g-glutamyl-S-[6-[(1E)-3-[(1R,2R)-1,2-dicarboxy-2-hydroxyethoxy]-3-oxo-1-propenyl]-2,3-dihydroxyphenyl]-L-cysteinyl- (9CI) |
| 683.225315 | Mp | C24H44O22 | Maltulose |

Supplemental table 2 : MS/MS identifications

| Yeast | Precurseur ion | Retention time (min) | Experimental fragments ; 15 eV Mode (-) | Spectrum matching | Elemental formula [M] | Annotation YMDB / Metlin / Metfrag |
| --- | --- | --- | --- | --- | --- | --- |
| SB1 | 149.0091 | 0.6 | 130.9988 | 1 | C4H6O6 | Tartaric acid |
| SB1 | 175.0611 | 2.2 | 113.061; 115.0401; 129.0558; 131.0715 | 4 | C7H12O5 | isopropylmalate |
| SB2 | 181.0507 | 1.5 | 117.0338; 119.0502; 132.0212; 133.0296;  134.0373; 135.0452; 137.0605; 163.0402 | 8 | C9H10O4 | Syringaldehyde / Homovanillic acid |
| SB1 | 189.0768 | 2.9 | 127.0763; 129.0557; 143.0712; 145.0873;  171.0661 | 5 | C8H14O5 | Diethyl malate |
| SB1 | 191.0199 | 0.7 | 111.0091; 129.0194; 130.9983; 143.0357;  147.0299; 154.9987; 173.0093 | 7 | C6H8O7 | citric acid |
| SB1 | 289.0719 | 2 | 123.0446; 125.025; 137.0241; 151.0403;  164.012; 165.0187; 167.0346; 179.0346;  187.04; 188.0485; 202.065; 203.0714;  205.0508; 221.082; 245.0821; 247.0627 | 16 | C15H14O6 | Catechin |
| Sc | 321.0053 | 3.6 | 125.9518; 134.0017; 147.0449; 151.0276;  152.0353; 162.0193; 164.0349; 176.0353;  179.0465; 194.046; 196.0502; 197.9743;  207.0413; 219.0417; 221.0561; 237.0516;  249.0517; 257.0336 | 18 | C14H6O8 | ellagic acid |
| SB1 | 325.0569 | 2.4 | 112.9874; 130.999; 134.0373; 149.0092;  149.0609; 178.0276; 193.0508 | 7 | C14H14O9 | Fertaric acid |
| LT | 333.0594 | 0.6 | 153.999; 241.0121; 242.0157; 259.0214 | 4 | C9H19O11P | 1-(glycero-3-phospho)-1-myo-inositol |
| SB2 | 389.1249 | 3.3 | 179.1082; 185.0612; 223.0983; 227.0716;  311.1024 | 5 | C20H22O8 | Piceid |
